# Views of male childhood cancer survivors on fertility preservation and restoration

**DOI:** 10.1530/RAF-25-0071

**Published:** 2025-11-19

**Authors:** Jillis van Maaren, Nienke de Graeff, Jaap G den Hartogh, Alied M van der Aa–van Delden, Marianne D van de Wetering, Heleen J H van der Pal, Leontien C M Kremer, Ans M M van Pelt, Callista L Mulder

**Affiliations:** ^1^ Reproductive Biology Laboratory, Amsterdam UMC, University of Amsterdam, Amsterdam, The Netherlands; ^2^ Amsterdam Reproduction & Development Research Institute, Amsterdam UMC, Amsterdam, The Netherlands; ^3^ Department of Medical Ethics & Health Law, Leiden University Medical Center, Leiden University, Leiden, The Netherlands; ^4^ The Novo Nordisk Foundation Center for Stem Cell Medicine (reNEW), Leiden Node, Leiden, The Netherlands; ^5^ Dutch Childhood Cancer Organization (Vereniging Kinderkanker Nederland), De Bilt, The Netherlands; ^6^ Princess Máxima Center for Pediatric Oncology, Utrecht, The Netherlands

**Keywords:** fertility restoration, testicular preservation, qualitative research, childhood cancer survivors

## Abstract

**Abstract:**

In this study, we aimed to uncover the perspectives of male childhood cancer survivors on parenthood, fertility preservation, and potential fertility restoration using their own cryopreserved testicular biopsy. We invited all young men eligible for this study, 27 male cancer survivors over the age of 16 who had undergone a testicular tissue biopsy in the past in Amsterdam, of whom five men (18.5% response rate) decided to participate. In semi-structured interviews, we discussed their views on parenthood, testicular cryopreservation, and potential fertility restoration, specifically through spermatogonial stem cell transplantation (SSCT). All data were pseudonymized before analysis via open coding using MAXQDA software. We found that all five participants (ages 17–24) had actively thought about family planning, and some expressed a very strong wish to experience parenthood, although they were aware of potential fertility issues related to their treatments. However, these participants reported that fertility issues had been minimally discussed at the late effects clinic during checkups, and they therefore had a limited understanding of the potential and restrictions of fertility restoration techniques such as SSCT. Overall, these participants displayed a high willingness to undertake additional steps to achieve biological parenthood and low levels of concern regarding the safety of SSCT. Autonomy and the opportunity of choice were determining factors in their underlying views. Perspectives and needs of these survivors, for whom a testicular biopsy was cryopreserved as a child, predominantly revolved around the importance of having autonomy over their fertility choices and being adequately informed to make those choices.

**Lay summary:**

Treatments for childhood cancer may lead to infertility. To safeguard the fertility of young male cancer patients, a testicular biopsy can be frozen that contains spermatogonial stem cells (SSCs), the basis for spermatogenesis. This study focused on SSCT, a new fertility restoration treatment. Through interviews with five male survivors (aged 17–24), we explored their experiences and opinions on fertility preservation and restoration. In general, participants were happy with their parents’ decision to freeze a biopsy. The freedom to choose was a key topic for them. They were willing to take the required steps to reach parenthood if desired. They had few concerns about the safety of SSCT but had questions about the chances of success. Their trust in science and healthcare was strong. However, in their experience, fertility was not sufficiently discussed during follow-up care. The results support doctors, former childhood cancer patients, and their parents concerning fertility after childhood cancer.

## Introduction

In recent years, the 5-year survival rates of children with childhood cancer have climbed to 81% (Botta *et al.* 2022). Given these improved survival rates, preventing and managing the late adverse effects of childhood cancer treatments has become increasingly important. As gonadotoxic cancer treatments can result in temporary or permanent infertility, these treatments are of particularly great consequence for later life (Mulder *et al.* 2021, Yumura *et al.* 2023).

To allow for fertility preservation in post-pubertal boys, a sperm sample can be collected and stored before these treatments if puberty has already progressed sufficiently. However, for pre-pubertal boys, the collection of genetic material is only possible through the cryopreservation of immature testicular tissue containing spermatogonial stem cells (SSCs). This involves taking a testicular biopsy, recommendations for which have been published in an international guideline (Mulder *et al.* 2021) and recent ESHRE good practice recommendations (ESHRE FP for Boys Working Group *et al.* 2025). Several experimental options to restore fertility using such biopsies are currently under development (Sanou *et al.* 2022), in line with growing opportunities for cancer survivors in general to access tailored fertility preservation and treatment (De Paola *et al.* 2025).

One of the possible future therapies to restore fertility using a cryopreserved testicular biopsy is spermatogonial stem cell transplantation (SSCT). SSCT involves the autotransplantation of spermatogonial stem cells, isolated from the cryopreserved testicular biopsy, back into the testis of the healthy adult childhood cancer survivor, with the aim of restoring spermatogenesis and the possibility of achieving natural conception (Sanou *et al.* 2022). The proof of concept for this therapy has been delivered in mice (Brinster & Avarbock 1994, Brinster & Zimmermann 1994, Kanatsu-Shinohara *et al.* 2003). In nonhuman primates, the transplantation of testicular cells has resulted in the formation of spermatids capable of fertilizing oocytes through intracytoplasmic sperm injection, leading to the formation of embryos up to a blastocyst stage (Hermann *et al.* 2012). Currently, however, this treatment is not yet clinically available for human cancer survivors. The current guidelines dictate that testicular biopsies should only be offered to children undergoing treatment protocols with a high chance of infertility (Mulder *et al.* 2021). Thus far, cryopreservation of testicular tissue has been performed for over 3,000 boys worldwide (Duffin *et al.* 2024). Generally, the biopsy is collected simultaneously with another procedure for which the boy has to undergo anesthesia, such as port-a-cath (PAC) placement. The collection of the biopsy itself is deemed safe when limited to an absolute maximum of 1 mL or 50% of testicular volume (Uijldert *et al.* 2017) and has a limited risk of side effects (Borgström *et al.* 2020, Kanbar *et al.* 2021, Braye *et al.* 2023).

Parental involvement in this decision is dependent on the patient’s age (Lampic & Wettergren 2019). For boys under the age of 12, the decision is entirely up to the parents or guardians/caretakers (from here on referred to as ‘parents’), whereas boys aged 12–16 are encouraged to be involved in the decision. Most parents opt for their child to undergo the testicular biopsy, despite the lack of a currently available therapy (Ginsberg *et al.* 2014, Wyns *et al.* 2015, Braye *et al.* 2019).

Little is known about how these decisions and their possible consequences later in life are viewed by the survivors themselves. In general, childhood cancer survivors can experience diverse psychosocial issues (Maas *et al.* 2024), including psychological distress related to future fertility (Green *et al.* 2003, Logan *et al.* 2019). In conversations with adolescent and young adult cancer survivors, the influence of cancer and its long-term side effects on relationships and fertility has emerged as a major theme (Kent *et al.* 2012, Stinson *et al.* 2015, Brown *et al.* 2016).

Previous studies specifically investigating perspectives on fertility preservation often include young adolescent boys who were able to store sperm samples at the time of treatment (Stein *et al.* 2014, Taylor & Ott 2016), which can be used in currently available fertility treatments. These studies emphasize the importance of fertility counseling and providing options for fertility preservation.

However, studies exploring the perspectives of survivors of pre-pubertal childhood cancer on fertility preservation through testicular biopsies are limited, and their perspectives may differ, considering the current lack of fertility restoration options. To our knowledge, there are currently no data on their views on fertility restoration techniques using their cryopreserved testicular tissues.

There are some data on decision factors among pre-pubertal boys who have been offered fertility preservation through tissue banking (Wyns *et al.* 2015). This study reported that adolescents exhibited higher anxiety about fertility, while children placed greater emphasis on their health over the ability to have a family, underscoring how outlooks may shift with age and development. However, more in-depth exploration of their views was limited, as this was a questionnaire-based study. In addition, since the majority of patient responses in this study came from children under the age of 12, it is likely that their parents helped to answer the questionnaires and thereby potentially shaped their responses.

Gathering additional insights is crucial for gaining a better understanding of the perspectives of this unique population of male survivors of pre-pubertal cancer. In a time when there is a strong call to pursue the clinical application of these techniques, the patients’ views cannot and must not be left behind.

The objective of this study was to reveal these perspectives through individual interviews with childhood cancer survivors from whom a testicular biopsy was obtained in a fertility preservation program in the Netherlands. These data can be used to improve the guidance these survivors receive and to better support current patients and their parents. In addition, these perspectives provide crucial information to physicians and researchers who aim to move forward with SSCT as a treatment to restore fertility and hope for these survivors’ future families.

## Methods

We performed a qualitative study using in-depth individual interviews with young adult childhood cancer survivors to investigate their perspectives on fertility preservation and restoration. Specifically, we investigated their perspectives on their (parents’) choice for cryopreservation; the testicular biopsy itself; the role of their cancer diagnosis and the existence of the cryopreserved biopsy on family planning; and their views on the use of this testicular biopsy for fertility restoration through SSCT.

### Ethical approval

The research protocol was submitted to the research ethics committee of the Amsterdam UMC before the initiation of the research. The research ethics committee determined that this study was exempt from the Medical Research Involving Humans Act (research proposal no. W22_131#22.174). Written informed consent was obtained from all participants of the study, before data collection. A data protection impact assessment was performed and registered in accordance with General Data Protection Regulation (GDPR) legislation.

### Participant recruitment and study design

Participants were considered eligible for inclusion if they were male childhood cancer survivors with a prepubertal cancer diagnosis, who had participated in the fertility preservation program (Uijldert *et al.* 2017) and had testicular tissue stored; were currently age 16 or older; and were fluent in Dutch. The presence of significant communication problems, for instance due to behavioral disorders, was an exclusion criterion for the study.

Eligible participants (*n* = 27) were identified through the collaboration of researchers at the Amsterdam UMC, where the patients originally received treatment and where the tissues are stored, with physicians of the Princess Máxima Center in Utrecht, where the patients receive long-term follow-up care.

Invitations were sent out by regular mail, and a reminder was sent after 4–6 weeks. All invited participants had previously received a consent letter for continued storage of their testicular tissue at the age of 16. All letters sent to the participants included postage-free envelopes for participants to send back their informed consent form. In addition, we asked them for their personal e-mail or phone number to set up follow-up appointments more easily. No other (medical) data were gathered other than the information participants provided within the study.

The original study aimed to hold multiple focus groups (for ages 16–18 and 19+) with 6–8 participants each, guided by a moderator at an off-site location. However, the invitations for these focus groups did not yield sufficient responses. Consequently, the study design was changed to individual semi-structured interviews. A second round of invitations also included a questionnaire as an alternative option to participate, as well as a short questionnaire asking men who did not want to participate to indicate their reason from a list of possible reasons, with an option to add their own (Table 1). Recruitment was stopped after two follow-up letters in the second round of invitations.

**Table 1:** Reasons to decline participation and frequency of reported reasons (*n* = 6/27 approached childhood cancer survivors). One respondent ticked multiple boxes (2, 8, and 9).

Reason to not participate or drop out		Frequency
1	The topic is too confronting to discuss	0
2	The time investment to participate in a focus group is too large	2
3	I prefer not to discuss these matters in a group	0
4	The time investment to participate in an interview is too large	0
5	I do not agree with my parents that the testicular biopsy was taken	0
6	I have had my fertility checked and expect no problems with having (future) children, therefore the topic is not relevant to me	1
7	I would like to remain childfree, therefore the topic is not relevant to me	0
8	I have not really considered the fact whether I would like to have children or not (it feels too far away), therefore the topic is not relevant to me	3
9	Other (participant response): *‘*I am currently in the final year of high school which requires my focus and energy*’*	1
10	Other (participant response): recent cancer reoccurrence	1

### Data collection

Semi-structured interviews were conducted by two researchers (JvM, CLM) using an interview guide (Supplementary Table 1 (see section on Supplementary materials given at the end of the article)), with one researcher (JvM) leading the interview and the second making notes and asking additional questions (CLM).

Participants were given the choice between an in-person or online (through Microsoft Teams) interview. In-person interviews were held at a location of the participant’s choice, either at a neutral meeting place close to their house or at the Amsterdam UMC. Meeting places were consistently selected to allow for private and undisturbed conversations. At the participant’s request, a meeting at their own house was also an option. All interviews were conducted in Dutch, the native language of both interviewers and all participants.

Individual interviews were scheduled to last one to two hours and consisted of three separate parts (Supplementary Table 1) related to the participant’s experience or perspective on i) family planning and medically assisted reproduction, ii) the collection of the testicular biopsy and their parents’ decision to do so, and iii) fertility restoration using SSCT. Between the second and third part of the interview, the participants were shown a short informational video about research on SSCT and its theoretical use in the clinic (Supplementary Table 2, Supplementary Fig. 1, and Supplementary Fig. 2). This video included a step-by-step illustration of the intended therapy, including an explanation of the need for cryopreservation before cancer treatments; fertility assessments in adult men; the collection of spermatogonial stem cells (SSCs) from the thawed biopsy and their propagation in culture; the autotransplantation of the cells into the testis; and the possible outcomes of the therapy, including full restoration of spermatogenesis and the potential for natural conception, or partial restoration of spermatogenesis necessitating follow-up with regular fertility treatments to achieve conception. In addition, a brief overview of milestones in research on SSCT was provided, including the proof of principle for the technique as established in the mouse model (Brinster & Avarbock 1994, Brinster & Zimmermann 1994, Kanatsu-Shinohara *et al.* 2003); the safety studies done on mice (Mulder *et al.* 2018, Serrano *et al.* 2021, 2023); and the continued work on the culture of human SSCs (Sadri-Ardekani *et al.* 2009, 2011). Participants could ask for additional clarification on these topics before continuing the interview.

All interviews were recorded with the permission of the participant, transcribed verbatim, and pseudonymized for further data analysis. Original audio recordings were destroyed when data analysis was finished. Pseudonymized research data (transcripts and analyses) are stored for 15 years from the date of collection.

### Data analysis

All collected data were handled and analyzed using pseudonyms. Transcribed interviews were coded following Strauss and Corbin’s approach to coding (Strauss & Corbin 1990). Two researchers (JvM, CLM) independently assessed an initial interview via open coding, from which coding rules were established and applied to the remaining interviews. Additional themes emerging from these interviews were added to the codebook. General themes were subsequently grouped into larger categories and applied to the transcripts. After coding the last interview, the researchers went back and recoded the first interviews to ensure that the codebook was consistently applied across all interviews in the study. Finally, the coded segments were investigated for occurrences, coverage, and configurations. During coding, transcript quotes were collected and used to illustrate the relevant themes. All coding was performed by both researchers previously involved in data collection. MAXQDA software (version 2022) was used for coding and analysis. For the purpose of writing this manuscript, relevant quotes were translated from Dutch to English in a manner reflecting their original intent as closely as possible. Any sensitive details or information that could allow identification of the participant were generalized to assure privacy.

## Results

### Participants

Out of 27 childhood cancer survivors who met our inclusion criteria and were invited, six agreed to participate in the study, one of whom later dropped out due to personal reasons (Table 1). Five other survivors declined to participate and listed their reasons for doing so (Table 1). These included practical reasons related to the timing or the time investment of the study, or the fact that fertility was not an issue for them at this time in their lives. Sixteen survivors did not reply. No questionnaires were returned to us.

Of the resulting five interviews, one interview was conducted online through Microsoft Teams, while the other four interviews were held in person either in a private room in a suitable meeting place close to their place of residence (twice), at the hospital (once), or at the participant’s own house (once). The participants’ ages ranged from 17 to 24 (average: 21.2) years old. The average age at treatment was 11 years (range: 9–13 years), and the average time since treatment was 10.2 years (range: 8–12 years).

### Perspectives

**Figure 1:**
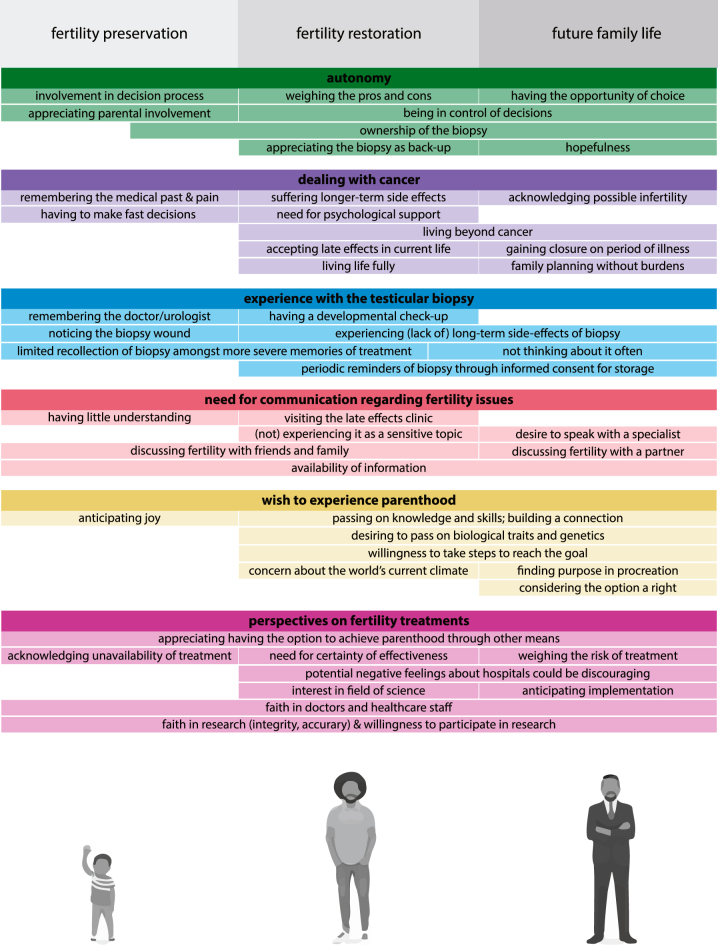
Graphical representation of collected perspectives on fertility issues across a survivor’s lifespan.

Six main themes were identified during the data analysis: living beyond cancer; the desire to experience parenthood; fertility preservation; fertility restoration; barriers and facilitators for future fertility treatments; and communication with physicians about fertility. Analysis of coded transcripts showed several emerging perspectives on past and present fertility issues, as well as participants’ hopes and desires for the future (Fig. 1). Representative quotes for the participants’ perspectives are collected in Table 2.

**Table 2:** Overview of participants’ quotes relating to specific themes. Edits to quotes to clarify meaning have been placed within brackets.

Theme	Subtheme	Quote
1. Living beyond cancer	The role of past cancer on future family planning	‘[My past cancer] does not play a role for me. I still have to deal with physiological symptoms, but I’ve learned to live with that’ ‘I have changed since my treatment, I have enjoyed life more fully. Maybe that contributes to me wanting to share that with future children as well’ ‘Every family has some genetic disorders, and every family, every person knows someone with cancer. So for me, that’s not a limitation [in biological parenthood]’
2. Desire to experience parenthood	A. Desire for a biological connection	‘If it really can’t happen in another way, I could adopt – but it’s very precious to me, to be able to share my family traits and genes, and to continue a line that goes back so far in time’ ‘I don’t want to bring a new child into this world. […] You could love an adopted child as much as your own blood. It wouldn’t matter that much to me – family is family’ ‘I think you rather exhaust your own options, before considering alternatives [like sperm donors or adoption]’
	B. Willingness to take steps to reach the goal of parenthood	‘As long as it’s possible to for me to have biological children of my own, I don’t really care what I have to do to make that happen’ ‘For me, the number of steps it takes is not a factor’
3. Perspectives on fertility preservation	A. Involvement in the decision process for fertility preservation	‘I was too sick; my parents had to make the decision for me. But I’m glad they did’ ‘I talked about it with them, but it was my parents’ decision. I was 12, I was too young to make that choice [about future kids]. I thought, “I’m a kid’’ ‘My parents have involved me in decisions throughout my treatment period and it was good to feel a sense of control. I think I was capable enough to do that, at that time. […] In that phase, before all the chemo, it felt like a minor decision to make’
	B. Past experience with the testicular biopsy	‘I remember the procedure, and the physician, but I don’t recall that the biopsy itself bothered me after. Although now, long-term it has had a few effects on the development and sensitivity’ ‘I can’t recall the procedure at all, because everything after was so much worse’
	C. Feelings about the existence of a currently stored biopsy	‘My parents’ decision was absolutely the right one. For me, it’s a way to still have children. And at that age I didn’t consider children, but looking back, if this is a means through which it’s still possible for me, it’s a great backup’ ‘I’m glad my parents made a choice. And I appreciate now having that choice too, whether I would use the biopsy or not, it’s good to know it’s there if I need it’
	D. Advantages and disadvantages to fertility preservation	‘For me, the risks are limited, so it’s worth those risks, because if you can’t have kids of your own, that can also do severe mental damage to yourself, so it’s nice to have it as a sort of insurance, and spread the risk’ ‘I think it’s a sense of assurance for yourself, so if you can’t have kids due to the chemotherapy, that you won’t think, ‘oh if only I had done it’, and then it’s too late’
4. Perspectives on fertility restoration	A. Feelings about fertility restoration	‘If I have to bring my feelings into words, it’s happiness, joy. I mean, it shows how far the research has come and how there is less and less time necessary to reach the goal’ ‘It’s more genius than I had expected. I think the way in which you hope it works, through natural conception, sounds like a very positive thing for many people, definitely from an emotional view’ ‘I think many people want to start a family…and if such an already profound disease takes that away from you, then it’s yet another thing the cancer takes from you even after having been treated. If this [therapy] then allows you to get that back, that’s amazing’
	B. Weighing advantages and disadvantages	‘I think I would want to do it, if I had enough certainty it would work’ ‘[…] At least you’re sure that if it doesn’t work, it doesn’t work. […] you wouldn’t want there to be a lingering gut feeling of, it could have been different and maybe better, even if your life is good at that time’ ‘You didn’t undergo the biopsy for nothing, and there is good research on it in mice. If it works, great, but if I don’t try it definitely won’t. So yes, bring it on’ ‘I think that any chance is some chance. If you’re infertile your odds are zero percent, so, I think every extra percent chance can help’
5. Barriers and facilitators for SSCT as a future fertility treatment	A. Barriers for choosing SSCT as a fertility treatment	‘Something could be missing [from this research], there is a lot we don’t know about the human body yet, so yeah maybe [SSCT] is safe to do already, maybe not yet, I can’t say’ ‘It may be an obstacle to undergo another procedure, and it may not be pleasant, but I would still do it, even while [the treatment] is in a testing phase’ ‘I reckon it won’t be a short trajectory, and it depends on how much of a medical whirlwind you’re capable of accepting, but, the treatment seems safe enough, that doesn’t seem an issue to me’
	B. Facilitators for choosing SSCT as a fertility treatment	‘I’m not sure I would mind being the first. It would definitely be more challenging than when there would be more data already. The costs [= risks, personal costs] would have to be pretty low, to accept a small chance of success, but on the other hand, I think you would mostly choose to do it, as long as the costs aren’t too high’ ‘I think if you want kids badly enough, the risks don’t matter. But, if you’re doubting whether to do it or not, I think it’s really important to have data on how well it works. You need to explain it really well in advance’ ‘Nothing ventured, nothing gained’
	C. Faith in research and healthcare	‘For me, call it faith or naivety, but I have faith that the research is done properly and that the risks would be acceptable’ ‘It seems to have been tested thoroughly, […] that the necessary steps in medical research have been taken. You don’t just get [approval for clinical testing], so I would assume it’s safe’
6. Communication with physicians about fertility	A. Communication during long-term follow up care	‘I can’t remember [the possibilities] being discussed specifically. I mentioned it myself a couple years ago, but then only discussed it briefly with my oncologist’ ‘I know [the biopsy] is there and my parents have told me some things about it, but it’s not entirely clear to me what [the possibilities with the tissues are]’ ‘No, it hasn’t been discussed at my check-ups. It’s also not that relevant to me at this time’ ‘At my first check-up, they asked if I had done [fertility preservation], but after that it hasn’t really come up’
	B. Barriers and needs for communication	‘I reckon it can be an uncomfortable topic to discuss. For me, I don’t mind, but… I think it’s up to the patient to initiate a conversation, if you want to discuss it’ ‘I think it would be good if it was mentioned at some moment in time after the chemo. […] and to provide some written information, like a flyer or e-mail afterward’ ‘With a doctor’s appointment, you usually remember only the first few minutes, and then it goes in one ear and out the other’ ‘I think that that part [relating to fertility restoration] should be a conversation between the [survivor] and the doctor and not between the parents and the doctor. […] The parents will always have a kind of power over their child, but, I think that choice [for fertility restoration] should be the child’s choice’

### Living beyond cancer

All participants still received regular check-up consultations. The cancer was fully in remission in four out of five participants, and the fifth was not receiving active treatments. All participants displayed a sense of ‘living beyond cancer’, as for them their illness did not play a significant role in their current life or in looking ahead toward the future (Table 2, Theme 1). In fact, one participant poignantly described the cancer’s impact, saying it had already taken so much – tainting good years with treatments and suffering. This increased both his sense of living to the fullest and his determination not to let cancer rob him of the opportunity of having a family, too.

Although some participants described the persistence of side effects of their cancer treatments (e.g. neuromuscular symptoms), they did not identify these as a burden holding them back in life. In general, the participants did not consider their medical past to be of importance in their desire and decisions to have children or not. One participant specifically mentioned his cancer to be nonheritable, and therefore not of relevance to him in the context of future offspring, whereas another considered his illness as something that could happen to anyone. The other participants did not mention potential heredity to be a consideration.

### Desire to experience parenthood

Most (4/5) participants had the desire for a future family with one or more biological children, with some of them describing this as a longstanding and deeply held wish. The desire to experience parenthood encompassed the ability to have a family life, the desire to pass on knowledge and skills to their offspring, and a general sense of joy. Some participants mentioned happy memories and experiences of their own childhoods and the desire to provide the same for a child of their own. If it were possible, these men strongly favored a biologically own child. Reasons for this included the ability to recognize themselves and family characteristics in them, and continuing the family name (Table 2, Theme 2, Subtheme A).

The fifth participant did not want a biologically related child but would consider adoption. For him, a sense of worry about bringing a child into the world in its current state strongly outweighed the need for a genetic connection. None of the participants were currently in the process of trying to conceive a child with a partner. However, those who desired genetic parenthood displayed a high willingness to undertake additional steps (i.e. fertility exams and treatments) to reach that goal in the future, if necessary (Table 2, Theme 2, Subtheme B).

### Fertility preservation

The intensity of the cancer treatments and hospital admissions strongly varied between participants, but for all of them the experience of the additional testicular biopsy was perceived as a minor matter among other more physically or emotionally demanding cancer-related procedures. All participants had been involved in the decision process, except in one case where the boy’s medical state at that time was too severe for him to be involved. For the other boys, their experiences of autonomy varied: some participated in the discussion while their parents made the final decision (ages at treatment: 9, 9, and 12), while another experienced complete autonomy in making his own choice (age at treatment: 13). In cases where parents made the final decision, these boys were happy to be involved but also recognized the importance of their parents having the final say, as they could not sufficiently oversee or appreciate the full scope of the consequences of their treatments at that time (Table 2, Theme 3, Subtheme A).

For all participants, the testicular biopsy was performed during another procedure (e.g., a port-a-cath placement), and most of them could not retrieve specific memories of this moment. Side effects of the biopsies were limited to some wound healing, except in one participant who at the time of the study still experienced some testicular tenderness and growth restriction in the biopsied testicle (Table 2, Theme 3, Subtheme B). Nevertheless, all interview participants reported that they agreed with their parents’ choice for fertility preservation through testicular biopsy (Table 2, Theme 3, Subtheme C). In addition, they would advise (parents of) other prepubertal cancer patients to opt for fertility preservation as well. The main reasons for this were the option to have the testicular tissue stored as a ‘backup’ in case of infertility, and the experienced absence of clear drawbacks of the procedure itself (Table 2, Theme 3, Subtheme D). None of the participants currently experienced any psychological distress due to the existence of the stored biopsy. For some, it was actually comforting to know that they had ownership of their biopsy and the option to keep it stored for potential future use. Generally, the biopsy was considered to provide the participants with the possibility of families of their own in case of treatment-induced infertility, reinforcing their sense of autonomy.

### Fertility restoration

The participants were unanimously and without hesitation in favor of any (medical) means to help infertile couples achieve a pregnancy. They contended that treatments such as *in vitro* fertilization (IVF) and intracytoplasmic sperm injection (ICSI) should be available for any couple who is unsuccessful in conceiving naturally, with the opportunity of choice as a decisive factor.

Knowledge of potential fertility restoration through the use of their testicular biopsy using SSCT was very limited in all participants. Most of them had a vague memory or idea of the procedure as it had been explained to them in the past, which broadly involved using the stored biopsy to isolate or create sperm. None of the participants had heard of the term ‘SSCT’ before, or of the related research that had been done. None of them reported having discussed the technique with any physicians at any point after their treatments.

When introduced to SSCT, the participants in our study were positive about the technical aspects and current studies on the use of SSCT as a means for fertility restoration (Table 2, Theme 4, Subtheme A). The treatment steps seemed logical to them, and they considered the established research findings in mice and in human cell cultures to be encouraging.

Again, having a choice and the autonomy to make one’s own decision was a prominent theme (Table 2, Theme 4, Subtheme B). Some participants indicated having discussed some of these matters with their parents but also mentioned a sense of discomfort when talking about these topics with them. Instead, one participant preferred to speak to a psychologist about issues surrounding fertility and sexuality.

### Barriers and facilitators for future fertility treatments

Participants with a wish for a biologically own child (4/5) displayed a large willingness to undertake steps to reach that goal, including fertility restoration through SSCT (Table 2, Theme 5, Subtheme A). Although one of them would be willing to sign up as a candidate straightaway, the other three displayed some hesitation relating to the uncertainty of the effectiveness and efficiency of SSCT. For them, having some data on the expected chance of success of the technique would be helpful in weighing the pros and cons to decide whether to undergo the procedure or not. However, all these participants voiced the opinion that if SSCT was the only chance for them to have children, they would take that opportunity even if the chances of success were very slim (Table 2, Theme 5, Subtheme B). One participant who did not desire children of his own was still in favor of the development of techniques such as SSCT to provide other survivors with the choice to have children.

Participants were also asked about the influence of potentially needing additional reproductive techniques following SSCT, such as IVF or ICSI, in case of insufficient sperm numbers for natural conception. None of them considered this to be a significant barrier to their decision for fertility restoration and mainly regarded it as just another step toward their goal of parenthood.

Most participants mentioned that the additional hospital visits and procedures could be a barrier for some patients, but for them personally they were not. Some mentioned they might expect some side effects related to the transplantation itself. The ultrasound-guided injection of cells back into the testis, as depicted in the informational video, was considered to perhaps give some testicular discomfort, much like taking the biopsy. Although the video also showed the use of laboratory-based cell culture to allow SSC propagation, the participants mentioned no concerns or fears specific to this process. They also did not mention any other perceived risks or fears, either for themselves as recipients or for their potential offspring conceived either naturally after SSCT or through the addition of a standard IVF or ICSI procedure. They showed a great amount of faith in the research done in this field, and to them the technical steps of the procedure seemed logical and largely harmless (Table 2, Theme 5, Subtheme C).

In the end, the central factor in all their perspectives on potential fertility restoration through SSCT was the wish for a child. If that wish was strong enough, it would outweigh everything else, with a sense of ‘nothing ventured, nothing gained’. Related to this was a perceived sense of regret and a sense of missed opportunity if they were unable to conceive and would not choose to at least attempt SSCT to fulfill their wish for a child.

### Physician communication relating to (in)fertility and fertility restoration

All participants reported good relationships with their former healthcare providers, including their oncologists and the andrologist involved in collecting the testicular biopsy. At the time of the interview, they all had regular checkups at the late effects clinic. Most participants retained a general understanding of the potential gonadotoxic nature of the cancer treatments, and therefore the potential worth of fertility preservation. At the same time, most also understood the experimental nature of fertility preservation at that time and the ongoing experimental nature of fertility restoration.

One participant recalled that the development of the biopsied testis had been checked, and some participants mentioned that their fertility preservation had been discussed at their first visit to the late effects clinic. However, none of the participants could recall that fertility restoration had ever been brought up or discussed by the physicians themselves throughout this period. For one participant, this meant that his original interpretation of the possibilities of fertility preservation and restoration, at age 9, had not been challenged since. During our conversations, he expressed a strong conviction that, no matter what, his biopsy could be used to restore his fertility and grant him the children that he strongly desires. In the other participants, awareness of the current experimental nature of fertility restoration through the use of a testicular biopsy was more pronounced (Table 2, Theme 6, Subtheme A).

Generally, there was a consensus that the initial discussion of fertility restoration should take place at the late effects clinic, after which referral to a specialist in fertility restoration was preferred. There were some differences in opinion as to whether the patient or the physician should start these initial conversations, which related to the degree to which the participants considered their fertility and sexuality to be a sensitive topic. Several participants mentioned that if they were at a life stage where fertility and family planning were important to them, they would not mind finding their way to the clinic and the help they needed themselves. This was especially pronounced in one of our slightly older participants, who had made the initial choice for fertility preservation himself. Others remarked that it would be beneficial for them if the topic of fertility was periodically brought up by the physician at the late effects clinic, as they themselves and perhaps others would be hesitant to share their thoughts and concerns unprompted.

Looking ahead, if SSCT were to become available as a means of fertility restoration, the participants would strongly appreciate written information about the technique itself, the chances of success, and the possible risks of treatment. Multiple participants remarked that the information provided in the doctor’s office is often only partially retained, and that they had trouble remembering all the specifics or details given to them at their times of treatment. These participants agreed that receiving written information would be beneficial because it would allow them to reread this information at home, and it would facilitate discussions with family and friends. However, one participant stated that such paperwork may also get lost easily or ignored when family planning was not a current issue (Table 2, Theme 6, Subtheme B).

Finally, one participant was of the opinion that conversations regarding fertility restoration should, in principle, always be between the physicians and the survivors themselves, notably without parental involvement. Though he was very appreciative of the involvement of his parents in the decisions regarding fertility preservation at the time, he emphasized the importance of avoiding parental influence on fertility restoration, and of autonomy and ownership of the testicular biopsy for himself (Table 2, Theme 6, Subtheme B).

## Discussion

This study aimed to uncover the perspectives of male childhood cancer survivors involved in a fertility preservation program on immature testicular tissue cryopreservation and fertility restoration, specifically using SSCT. Through in-depth individual interviews with five survivors whose testicular biopsies have been preserved, we were able to document their experiences thoroughly, helping to bridge the current knowledge gap regarding this patient group. Their perspectives on various topics brought forward an overarching theme of reproductive autonomy, which played a pivotal role in decisions about fertility preservation, in contemplating fertility restoration, and in communicating about fertility issues. These perspectives, albeit from a small number of men, are of importance to fertility preservation practice and to ongoing research on fertility restoration in its current pre-clinical phase.

### Deliberating fertility preservation

Encouragingly, all the participants in this study were very happy with the decision to collect the testicular biopsy at the time of treatment. This mirrors the families’ feelings of having made the right decision retrospectively (Ginsberg *et al.* 2014), with the main reported reason being the severe negative impact that infertility might have on the future quality of life (Ginsberg *et al.* 2014, Wyns *et al.* 2015).

Age played a role in the boys’ involvement in this decision, as did the severity of their medical status at the time of treatment. However, individual differences were also apparent in either experiencing this moment as an opportunity for autonomy and making one’s own (informed) choices, or experiencing it as a moment in which they, as a child, could thankfully rely on their parents to help them. Interestingly, all participants had a clear and positive recollection of the andrologist who guided them in this decision and performed the testicular biopsy. This illustrates the strong influence and impressions that physicians can have on their patients and how important it is to guide them through their tough times as well as possible. The fertility counseling, as experienced by our participants, is in line with existing guidelines that strongly recommended informing cancer patients and their families about the possibility of infertility (Mulder *et al.* 2021). Simultaneously, the difficulty in timing these discussions due to the severity of the illness, previously described by parents and patients (Taylor & Ott 2016) as well as physicians (Lampic & Wettergren 2019), was also mentioned by some of our participants.

### Creating choice through fertility restoration

For these five men, their past cancer played little to no role in their choices in their current lives. In contrast to previous findings regarding fertility concerns in cancer survivors (Benedict *et al.* 2016), none of the participants reported a concern with passing on a genetic risk for cancer. The cancer also did not negatively affect their desire to have a family, which for some seemed even increased by their treatment period. This matches previous findings where survivors reported a heightened desire to have children, influenced by their experiences with cancer (Schover 2005, Stein *et al.* 2014, Brown *et al.* 2016).

While the lack of an available clinical application for the biopsy has been reported to be a barrier in deciding on fertility preservation (Ginsberg *et al.* 2014), none of our participants expressed such concerns. Despite knowing the experimental nature of fertility treatments using the biopsy, they generally expected such procedures to be available to them in the future.

In our study, SSCT was generally perceived as simply another fertility technique, not unlike what they knew of IVF, to achieve their goal of parenthood. In that regard, the collection of the testicular biopsy provided an opportunity to choose fertility restoration in the future, and they were glad to have that choice.

The participants in this study shared a strong faith in the people involved in fertility research and healthcare, similar to previous findings of the levels of trust placed in medical professionals providing established fertility treatments (Perrotta & Hamper 2023) but also novel technologies (Daal *et al.* 2024). Especially in going forward with SSCT to clinical trials, clear and honest communication should be prioritized to mitigate the emergence of unrealistic expectations or therapeutic misconception, and to encourage patients to communicate their concerns with their healthcare providers.

### Communicating fertility issues

Communication regarding fertility preservation and restoration should be periodically evaluated and adjusted where necessary according to the latest findings in (pre-)clinical studies on fertility preservation and restoration. Our study showed that these participants had a good understanding of the intention of fertility preservation through the collection of the testicular biopsy, matching previous findings (Drechsel *et al.* 2024). However, the current status of options for fertility restoration through the use of this biopsy was unclear to these participants. Although the experimental nature of fertility restoration at the time of collection of the biopsy was largely known, multiple participants considered the biopsy as a backup, providing them with some assurance of a chance for parenthood. In one participant in particular, a false sense of security prevailed, as his interpretation of the options that were explained to him at the time of fertility preservation had not been corrected. This may demonstrate an age-dependent comprehension of fertility counseling at the time of the biopsy, as previously reported by Wyns *et al.*, who found that only 33.3% of boys under the age of 12 were able to understand the provided information, in contrast to 90.9% of boys over 12 years old (Wyns *et al.* 2015). Moreover, these findings underscore the importance of developmentally appropriate conversations and repeating such information over time, providing additional guidance relevant to the survivors’ developmental phase (Kuntz *et al.* 2024).

In the experience of this study’s participants, the topic of fertility (restoration) was minimally discussed at the late effects clinic during their visits. This could indicate a mismatch between the current guidelines and the patients’ experiences. Participation in our study could itself be considered a potential bias. However, participants also mentioned limited conversations in their past visits, which may have various causes.

First, the extent to which these conversations took place may depend on the survivor’s own interest at that time, which can rapidly change during adolescence. Therefore, needs could strongly change in the time between appointments, which may be too far apart for some.

Second, as fertility is only one of a range of late effects that are discussed during a consultation, there is the possibility that this topic does not receive enough attention. This could especially be true for some patients who, like some of our participants, experience fertility as a sensitive topic.

Finally, whether their parents are present at these consultations could also have an important influence. The presence of parents could affect the choice of conversation topics or discourage children from raising fertility issues if, like some of our participants, they are not comfortable discussing these matters with their parents. Conversely, physicians might also avoid in-depth discussion of fertility topics when they perceive them to cause discomfort for the patients and their parents. However, this discomfort has been reported to be overestimated (Ginsberg *et al.* 2008, Stein *et al.* 2014).

Currently, when survivors at the late effects clinic have fertility-based questions, a referral to a nearby fertility specialist is recommended to discuss fertility and potential issues more thoroughly. This would provide survivors with an opportunity to voice their concerns regarding these matters and to improve awareness and understanding of the (im)possibilities of fertility restoration. In addition, this helps align care for survivors who are either still seen by their pediatrician or by a physician at the late effects clinic, which may also impact the way in which fertility issues are discussed.

Specifically, offering written patient information (e.g., digitally or by leaflets) on the topics of fertility issues and potential fertility restoration could be recommended to improve recall of the provided information.

### Strengths and limitations

A major strength of this study was the access to a unique cohort of men who survived cancer and who had participated in a fertility preservation program cryopreserving their immature testicular tissue as a child. The project team consisted of expert researchers in the field of SSCT as well as physicians involved in the long-term care of cancer survivors. The additional input of an ethicist, psychologist, and affiliate of the patient organization was instrumental in formulating cohort-appropriate communication.

However, although a reasonable percentage of men (18.5%) accepted our invitation to participate in the study, its small sample size remains a limitation, as does the self-selection of participants. We anticipated this might correlate with selection bias, as men with negative hospital experiences, or with severe late effects, as well as men without the desire to become fathers, might be less inclined to participate. However, multiple participants reported rather intense medical experiences related to their cancer treatments. Similarly, one of the participants did not wish to have any biologically related children in the future. Although one man dropped out of the study due to cancer recurrence, another man who was not free of cancer at the time of the study, though without treatment, was still willing to participate. Five young men who declined to participate cited practical reasons or felt the topic was not yet relevant to them. Interestingly, we could not identify age as a limiting factor, as our youngest participant was, at 17 years old, already clear in his desire for a family.

Due to low recruitment rates, the study design was adjusted from focus groups to individual interviews. However, this change ultimately proved to be more effective, offering greater flexibility in scheduling, minimizing travel time for participants, and allowing them to speak freely about sensitive topics. For future studies that may combine cohorts internationally to acquire a larger number of participants, focus groups may be a suitable approach, although language and cultural barriers should be taken into account when undertaking such an endeavor.

Notably, qualitative research always allows for researcher bias through the types of questions asked as well as through researcher positionality in data collection and analysis. In this study, this was mitigated by the continuous involvement of two researchers as well as by relying on an interview guide for the interviews. An additional strength of this interview design was created by the pre-recorded video to provide participants with an explanation of SSCT, which improved uniformity.

The knowledge obtained within this study could also extend to patient groups included in the fertility preservation program due to gonadotoxic treatment of non-cancerous pathologies. Currently, pre-pubertal sickle cell patients are also offered fertility preservation in the form of a testicular biopsy (Duffin *et al.* 2024). However, these patients may face different challenges during their treatments, warranting additional research on their views.

## Conclusion

This study provided a unique platform to male childhood cancer survivors who have undergone testicular tissue cryopreservation to share their in-depth perspectives on decisions regarding fertility preservation and potential fertility restoration through SSCT.

The majority of the participants in this study shared a strong wish for a biological child. However, their experiences in the late effects clinic included limited counseling on fertility issues, especially fertility restoration options. Nevertheless, these participants demonstrated a high willingness for additional fertility treatments, including SSCT, and for most, the benefit of potential biological parenthood outweighed the perceived risks. For them, cryopreservation of their testicular tissue provided a sense of reproductive autonomy and the opportunity for choice in their family planning.

The findings from this small cohort of young male survivors may strengthen parents’ resolve to involve their children in the decision for fertility preservation. Still, the views of survivors or their parents who decided to opt out are of interest, as they would be expected to experience different barriers, such as decision regret (Wyns *et al.* 2015, Kuntz *et al.* 2024). Our findings may also help physicians counsel their patients and guide them through their fertility concerns throughout the trajectory of care, from childhood into adulthood. Fertility issues should be actively raised during consultations, as advised by international and national guidelines, in a manner that matches the survivors’ developmental stage and cognition at that time. Additional recommendations include discussing whether the presence of their parents is desirable during these conversations and developing specific written patient information.

Our data indicate that these survivors support the development of fertility restoration treatments to build a future where the cancer’s hold on people’s lives is reduced, and where survivors who desire parenthood can reclaim their fertility and the fullness of their lives.

## Supplementary materials



## Declaration of interest

The authors declare that there is no conflict of interest that could be perceived as prejudicing the impartiality of the work reported.

## Funding

This study was funded by a ZonMW VIMP grant (grant no. 01160462210002).

## Author contribution statement

This study was designed through a specially assembled consortium, including members of the Amsterdam UMC, where the patients originally received treatment and where the tissues are stored (AMMvP, CLM, JvM); physicians of the Princess Máxima Center, where the patients receive long-term follow-up care (HJHvdP, LCMK, MDvdW); a psychologist associated with the long-term follow-up care at the Princess Máxima Center (AMvdA-vD); an ethicist with a background in bioethics and specific expertise in emerging biomedical technologies and therapies, such as stem cell treatments (NdG); and a representative of the Dutch patient association for children with cancer and their parents (Vereniging Kinderkanker Nederland; JGdH). Interviews were held by JvM and CLM, who also performed data analysis. JvM drafted the main body of the manuscript, which was concurrently critically revised by all other authors. All authors provided final approval of the version to be published and agreed with all aspects of the work, including the accuracy or integrity of any part of this work.

## Data availability

The data used in this article will be shared upon reasonable request to the corresponding author.

## References

[bib1] Benedict C, Shuk E & Ford JS 2016 Fertility issues in adolescent and young adult cancer survivors. J Adolesc Young Adult Oncol 5 48–57. (10.1089/jayao.2015.0024) 26812452 PMC4779291

[bib2] Borgström B, Fridström M, Gustafsson B, et al. 2020 A prospective study on the long-term outcome of prepubertal and pubertal boys undergoing testicular biopsy for fertility preservation prior to hematologic stem cell transplantation. Pediatr Blood Cancer 67 e28507. (10.1002/pbc.28507) 32649054

[bib3] Botta L, Gatta G, Capocaccia R, et al. 2022 Long-term survival and cure fraction estimates for childhood cancer in Europe (EUROCARE-6): results from a population-based study. Lancet Oncol 23 1525–1536. (10.1016/s1470-2045(22)00637-4) 36400102

[bib4] Braye A, Tournaye H & Goossens E 2019 Setting up a cryopreservation programme for immature testicular tissue: lessons learned after more than 15 years of experience. Clin Med Insights Reprod Health 13 1179558119886342. (10.1177/1179558119886342) 31798308 PMC6868573

[bib5] Braye A, Delgouffe E, van der Werff Ten Bosch J, et al. 2023 Gonadal development and function after immature testicular tissue banking as part of high-risk gonadotoxic treatment. Pediatr Blood Cancer 70 e30370. (10.1002/pbc.30370) 37150973

[bib6] Brinster RL & Avarbock MR 1994 Germline transmission of donor haplotype following spermatogonial transplantation. Proc Natl Acad Sci U S A 91 11303–11307. (10.1073/pnas.91.24.11303) 7972054 PMC45219

[bib7] Brinster RL & Zimmermann JW 1994 Spermatogenesis following male germ-cell transplantation. Proc Natl Acad Sci U S A 91 11298–11302. (10.1073/pnas.91.24.11298) 7972053 PMC45218

[bib8] Brown MC, Pearce MS, Bailey S, et al. 2016 The long-term psychosocial impact of cancer: the views of young adult survivors of childhood cancer. Eur J Cancer Care 25 428–439. (10.1111/ecc.12380) 26391419

[bib9] Daal Mv., de Kanter A-FJ, Custers RJH, et al. 2024 Patient, parent and professional expert perspectives on personalized regenerative implants: a qualitative focus group study. Regen Med 19 393–406. (10.1080/17460751.2024.2386214) 39222046 PMC11370919

[bib10] De Paola L, Napoletano G, Gullo G, et al. 2025 The era of increasing cancer survivorship: trends in fertility preservation, medico-legal implications, and ethical challenges. Open Med 20 20251144. (10.1515/med-2025-1144) PMC11826245 39958979

[bib11] Drechsel KCE, Ijgosse IM, Slaats S, et al. 2024 Fertility-preserving treatments and patient- and parental satisfaction on fertility counseling in a cohort of newly diagnosed boys and girls with childhood hodgkin lymphoma. Cancers 16 2109. (10.3390/cancers16112109) 38893227 PMC11171249

[bib12] Duffin K, Neuhaus N, Andersen CY, et al. 2024 A 20-year overview of fertility preservation in boys: new insights gained through a comprehensive international survey. Hum Reprod Open 2024 hoae010. (10.1093/hropen/hoae010) 38449521 PMC10914450

[bib14] Ginsberg JP, Ogle SK, Tuchman LK, et al. 2008 Sperm banking for adolescent and young adult cancer patients: sperm quality, patient, and parent perspectives. Pediatr Blood Cancer 50 594–598. (10.1002/pbc.21257) 17514733

[bib13] ESHRE FP for Boys Working Group, Mitchell RT, Eguizabal C, Goossens E, et al. 2025 ESHRE good practice recommendations on fertility preservation involving testicular tissue cryopreservation in children receiving gonadotoxic therapies. Hum Reprod 40 1391–1431. (10.1093/humrep/deaf106) 40574354 PMC12314154

[bib15] Ginsberg JP, Li Y, Carlson CA, et al. 2014 Testicular tissue cryopreservation in prepubertal male children: an analysis of parental decision-making. Pediatr Blood Cancer 61 1673–1678. (10.1002/pbc.25078) 24777742 PMC4676076

[bib16] Green D, Galvin H & Horne B 2003 The psycho-social impact of infertility on young male cancer survivors: a qualitative investigation. Psychooncology 12 141–152. (10.1002/pon.622) 12619146

[bib17] Hermann BP, Sukhwani M, Winkler F, et al. 2012 Spermatogonial stem cell transplantation into rhesus testes regenerates spermatogenesis producing functional sperm. Cell Stem Cell 11 715–726. (10.1016/j.stem.2012.07.017) 23122294 PMC3580057

[bib18] Kanatsu-Shinohara M, Ogonuki N, Inoue K, et al. 2003 Long-term proliferation in culture and germline transmission of mouse male germline stem cells. Biol Reprod 69 612–616. (10.1095/biolreprod.103.017012) 12700182

[bib19] Kanbar M, de Michele F, Giudice MG, et al. 2021 Long-term follow-up of boys who have undergone a testicular biopsy for fertility preservation. Hum Reprod 36 26–39. (10.1093/humrep/deaa281) 33259629

[bib20] Kent EE, Parry C, Montoya MJ, et al. 2012 “You’re too young for this”: adolescent and young adults’ perspectives on cancer survivorship. J Psychosoc Oncol 30 260–279. (10.1080/07347332.2011.644396) 22416959 PMC4560238

[bib21] Kuntz H, Santucci J, Butts S, et al. 2024 Determinants of decision regret regarding fertility preservation in adolescent and young adult cancer survivors: a systematic review. J Adolesc Young Adult Oncol 13 726–737. (10.1089/jayao.2023.0191) 38717190

[bib22] Lampic C & Wettergren L 2019 Oncologists’ and pediatric oncologists’ perspectives and challenges for fertility preservation. Acta Obstet Gynecol Scand 98 598–603. (10.1111/aogs.13551) 30714120

[bib23] Logan S, Perz J, Ussher JM, et al. 2019 Systematic review of fertility-related psychological distress in cancer patients: informing on an improved model of care. Psychooncology 28 22–30. (10.1002/pon.4927) 30460732

[bib24] Maas A, Maurice-Stam H, Feijen EAM, et al. 2024 Risk and protective factors of psychosocial functioning in survivors of childhood cancer: results of the DCCSS-LATER study. Psychooncology 33 e9313. (10.1002/pon.9313) 39358839

[bib25] Mulder CL, Catsburg LAE, Zheng Y, et al. 2018 Long-term health in recipients of transplanted in vitro propagated spermatogonial stem cells. Hum Reprod 33 81–90. (10.1093/humrep/dex348) 29165614 PMC5850721

[bib26] Mulder RL, Font-Gonzalez A, Green DM, et al. 2021 Fertility preservation for male patients with childhood, adolescent, and young adult cancer: recommendations from the PanCareLIFE consortium and the international late effects of childhood cancer guideline harmonization group. Lancet Oncol 22 e57–e67. (10.1016/S1470-2045(20)30582-9) 33539754

[bib27] Perrotta M & Hamper J 2023 Patient informed choice in the age of evidence-based medicine: IVF patients’ approaches to biomedical evidence and fertility treatment add-ons. Sociol Health Illn 45 225–241. (10.1111/1467-9566.13581) 36369731 PMC10100272

[bib28] Sadri-Ardekani H, Mizrak SC, Van Daalen SKM, et al. 2009 Propagation of human spermatogonial stem cells *in vitro*. JAMA 302 2127–2134. (10.1001/jama.2009.1689) 19920237

[bib29] Sadri-Ardekani H, Akhondi MA, van der Veen F, et al. 2011 *In vitro *propagation of human prepubertal spermatogonial stem cells. JAMA 305 2416–2418. (10.1001/jama.2011.791) 21673293

[bib30] Sanou I, van Maaren J, Eliveld J, et al. 2022 Spermatogonial stem cell-based therapies: taking preclinical research to the next level. Front Endocrinol 13 850219. (10.3389/fendo.2022.850219) PMC9013905 35444616

[bib31] Schover LR 2005 Motivation for parenthood after cancer: a review. J Natl Cancer Inst Monogr 34 2–5. (10.1093/jncimonographs/lgi010) 15784811

[bib32] Serrano JB, van Eekelen R, de Winter-Korver CM, et al. 2021 Impact of restoring male fertility with transplantation of in vitro propagated spermatogonial stem cells on the health of their offspring throughout life. Clin Transl Med 11 e531. (10.1002/ctm2.531) 34709748 PMC8506643

[bib33] Serrano JB, Tabeling NC, de Winter-Korver CM, et al. 2023 Sperm DNA methylation is predominantly stable in mice offspring born after transplantation of long-term cultured spermatogonial stem cells. Clin Epigenetics 15 58. (10.1186/s13148-023-01469-x) 37029425 PMC10080964

[bib34] Stein DM, Victorson DE, Choy JT, et al. 2014 Fertility preservation preferences and perspectives among adult Male survivors of pediatric cancer and their parents. J Adolesc Young Adult Oncol 3 75–82. (10.1089/jayao.2014.0007) 24940531 PMC4048980

[bib35] Stinson JN, Jibb LA, Greenberg M, et al. 2015 A qualitative study of the impact of cancer on romantic relationships, sexual relationships, and fertility: perspectives of Canadian adolescents and parents during and after treatment. J Adolesc Young Adult Oncol 4 84–90. (10.1089/jayao.2014.0036) 26812556

[bib36] Strauss A & Corbin JM 1990 Basics of Qualitative Research: Grounded Theory Procedures and Techniques. Thousand Oaks, CA, US: Sage Publications, Inc.

[bib37] Taylor JF & Ott MA 2016 Fertility preservation after a cancer diagnosis: a systematic review of adolescents’, parents’, and providers’ perspectives, experiences, and preferences. J Pediatr Adolesc Gynecol 29 585–598. (10.1016/j.jpag.2016.04.005) 27108230 PMC5903553

[bib38] Uijldert M, Meißner A, de Melker AA, et al. 2017 Development of the testis in pre-pubertal boys with cancer after biopsy for fertility preservation. Hum Reprod 32 2366–2372. (10.1093/humrep/dex306) 29040511

[bib39] Wyns C, Collienne C, Shenfield F, et al. 2015 Fertility preservation in the male pediatric population: factors influencing the decision of parents and children. Hum Reprod 30 2022–2030. (10.1093/humrep/dev161) 26141713

[bib40] Yumura Y, Takeshima T, Komeya M, et al. 2023 Long-term fertility function sequelae in young male cancer survivors. World J Mens Health 41 255–271. (10.5534/wjmh.220102) 36593712 PMC10042651

