# Supplementary material for: Views of male childhood cancer survivors on fertility preservation and restoration
Source: Reprod Fertil. 2025 Nov 19;6(4):e250071. doi: 10.1530/RAF-25-0071 (PMC12630533; doi:10.1530/RAF-25-0071)
Supplement: Supplementary file 1 [file supplementary_materials.pdf]

Supplementary table 1 - Interview guide for individual interviews.

Part	Topic	Question/information	Remark for interviewer	Checklist	Estimated time (min)
**Welcome, Reception**	n/a	Where we sit, offer cup of coffee, etc.	Neutral reception.		5-10
				√	
**Introduction about interview; explain set-up**	Explanation about the study	Explanation about the study aims			5-10
		Check if patient information folder was sufficiently clear? + check participant's signature on informed consent.			
		Explain role of both interviewers (timing, taking notes). NB Note that we as researchers are not doctors and we do not have access to their medical files.			
		Note that taking a break or deciding to stop is possible at any time. Participant can choose not to answer questions, and are free to share however much they want (or don't want).			
	Explanation about the interview itself	We will make an audio recording. These will be transcribed using a pseudonym, and audio recordings will be destroyed after use.			
		The interview consists of three parts and will take approximately 1 to 2 hours.			
		Explain the three parts.			
		Thanks for your participation in advance.			
				√	
**Part 1**	Questions about having kids in general (family building).	People can have different reasons to want, or to not want, children in the future. These could be their biologically own children, or adopted children or stepchildren.<br>❖ Have you ever thought about whether you might like to have children, in the future?<br>❖ (How old are you now?)			10-20
		❖ What factors influence your choice, when thinking about wanting children or not?	(elaborate on the given answer – why does this play an important role for you?)		

		❖ What is the influence of your past cancer treatments on our thoughts about (not) having a family?			
		❖ If you're currently in a (long-term) relationship: how do you experience talking to them about these topics? Or, what do you think about when anticipating such conversations in the future?	/what's a partner's influence on your thoughts to want children or not?		
	Opinions about biologically own children	How important is it to you, that your children are genetically related to you (biologically your own)?<br>(which is to say: not adopted, no stepchildren, and not conceived through a sperm donor).	Can skip this question if participant is resolute in their wish to not have children.		
		❖ Which factors contribute to this?			
		❖ / Can you indicate how important this is to you, on a scale from one to five where 1 is 'very unimportant' and 5 is 'very important'?	Ask alternative question if participant has difficulties specifying his answers.		
	Natural conception versus fertility treatments	Getting pregnant is not always possible for everyone. If a couple can't conceive via natural conception, the hospital can offer various fertility treatments. One example is IVF, which involves fertilization of the egg cell with the sperm in the lab, after which the resulting embryo is transferred to a woman's uterus, possibly a surrogate.<br><br>What are your views **in general**, so not necessarily for yourself, on the use of these kinds of fertility treatments?	If the participant finds this a difficult question, try phrasing it in a statement: "Some people think that if you can't conceive in a natural way, you shouldn't want to try in any other way at all, even if that's available. What are your thought on that?"		
		Possible follow-up question to invite sharing more on this topic:<br>Do you know people in your environment, that have had to deal with this? What kind of impression has that made on you?			
	Afronding	End of part 1;<br>❖ Are there other things you would like to share on this subject, before going to part 2?			

				✓	
**Part 2**	Questions about their perspectives on collecting the testicular biopsy (fertility preservation)	Introduction: cancer treatments (irradiation or chemotherapy) can do damage to the stem cells in the testis, which are responsible for formation of sperm cells. That's why, prior to such treatments, it can be decided to take a piece of the testis (a biopsy) and freeze it, to safely store the stem cells. Thankfully this isn't always necessary as many men who have been treated for cancer retain their fertility.<br>In the past, your parents have chosen to have the testicular biopsy done and cryopreserved. These following questions are about your perspectives on that time and how you view it now, looking back.			10-20
	The parents' decision	Your parents have made this decision for you.<br>❖ Can you recall whether you were involved in the decision?<br>❖ What was your age at that time?			
		❖ Which memories do you have from the procedure itself (the collection of the biopsy)?			
		It's quite a while ago now.<br>❖ How do now about your parents decision now, to have that biopsy taken?<br>(Do you agree with that choice?)	*If the participants finds this a difficult question, consider asking:*<br>"This biopsy is now stored (in the freezer). How does that feel, what kind of emotions or thoughts does that invoke in you?"<br>Or<br>"Have you ever again thought about the fact that that tissue is still stored?"		
		Follow-up question: which factors play a role in this for you/ which advantages and disadvantages do you see with this decision?	*If the participant needs another prompt, consider:* "does it give you a positive feeling that the tissue is present as a backup? Or, does it give you a strange feeling, or one of comfort?"		

	Information/knowledge that the participant already has about fertility restoration, using the biopsy.	Perhaps you have received information, in the past or at a later time, about the possibilities and impossibilities of using the biopsy for future fertility treatments.<br>❖ Which information have you received?<br>❖ Who gave you this information?			
	Primary thoughts/feeling on the use of freezing a testicular biopsy	Perhaps it's a bit of a difficult question – but looking back on this:<br>❖ Would you, yourself, with your current experiences and knowledge, advise someone else/another cancer patient to have the testicular biopsy done and stored, if the option is there?			
		❖ Why yes, or why not? (what factors into this?)			
				✓	
**Video**	Informative video about a possible future therapy (SSCT)	Introduction video clip			
	Video	Show video, parts 1 + 2			10
	Ask whether the information is clear	It's quite a lot of information to digest. We would like to ask you some questions about your thoughts and feelings about this therapy; but first we want to answer any questions you might have about these topics as portrayed in the video.<br>❖ Which parts of the explanation are currently still unclear to you?<br>❖ //which questions do you have, about the explanation of..<br>○ ...performing the therapy<br>○ …the research on this therapy			5

				✓	
**Part 3**	Questions about the possible use of the testicular biopsy (fertility restoration)	Introduction: in this part we would like to ask you some questions, about a possible future therapy, spermatogonial stem cell therapy, where the frozen piece of testis tissue could be used as a fertility treatment.			10-20
	Primary thoughts/feelings about the therapy.	We would like to know your thoughts on these matters.<br>❖ Had you already heard of this therapy, did you know of it?			
		❖ Which thoughts come up, when hearing about this therapy?			
		❖ What feelings arise, when hearing about this therapy? (What feelings does this elicit in you?)			
	Thoughts/feelings about the research done on this therapy (effectiveness, safety)	We just gave you some information about the research that has been done on this therapy.<br>❖ In your opinion, has there been sufficient research into this therapy to start applying it in humans?	*If the participant needs more prompts, consider:*<br>Do you think, that there is sufficient support for the *effectiveness* of the therapy?<br><br>Do you think, that there is sufficient support for the *safety* of the therapy?		
		If not – what are you mainly concerned about/ are there other matters you're also concerned about?<br><br>If yes – can you indicate, what's the main factor that gives you the feeling (that it has been sufficiently researched)?			
	Therapy outcomes	What if spermatogonial stem cell transplantation is possible as a fertility treatment;<br>❖ How important is it to you, that after treatment you are sure of restored fertility? ('guarantee of success')?	*That is to say:* Would you not consider the treatment, unless you're certain that it would make you fertile? Or is a (small) chance of success enough, to want the therapy?		
		❖ How important is it to you, that after the treatment you are able to conceive naturally (that is, via sex with a female partner)?	*Alternatively/additionally:*<br>If the therapy leads to formation of new sperm cells, but it's still necessary to do an additional fertility treatment (like IVF) to conceive a		

			pregnancy – would you still consider the therapy, or not?		
	Secondary thoughts and feelings about the therapy, 'outside of yourself'	At this time, spermatogonial stem cell therapy is not yet offered in the clinic. In the future, that might be possible, when someone would like biological children of their own, but is unable to do so due to cancer-treatment induced infertility.<br><br>A similar question to before – perhaps a bit abstract, but:<br>❖ Would you, from your current experience and knowledge, advise another to do the transplantation (or not)?<br>❖ What are your reasons to do so?	What advice might you receive from a friend?		
	Wrap-up part 3	This is the end of part 3 of this interview.<br>❖ Are there other things you might like to mention or discuss about this possible therapy?	Does this invoke any other questions or thoughts in you, that you would like to mention or discuss?		
				✓	
**Wrap-up**	Wrap-up	We have now finished all three parts of the questions of this interview: about your general perspectives on family building; having children; collecting the testicular biopsy; and the future possible therapy to restore fertility. That brings us to the end of this interview.	(summing up the parts may help the participant look back on the conversation and consider any questions they might have).		5-20
	Handling final questions	We have a few questions remaining, to wrap up.<br>❖ First off: are there topics of issues that you would like to still mention or discuss yourself?			
		❖ Secondly: do you currently have any questions about what you talked about with us today, and what you have heard about spermatogonial stem cell transplantation?			

		❖ Finally, we would like to ask you, if you have any questions about the research itself?			
	Thanks	Thanking participant for their participation + practical information about travel cost reimbursement; hand over small gift as token of our gratitude.			

Supplementary Table 2 – Script for informational video on SSCT, for use during interview.
Images used (‘image on screen’) are added as Supplementary Figure 1 and Supplementary Figure 2.

Section	Text	Image on screen
Introduction (1)	In this video we would like to inform you about a possible future therapy to treat infertility in men, who have become infertile due to cancer treatments at a young age (before puberty).	Amsterdam UMC Logo;<br>Amsterdam UMC
Introduction (2)	This therapy is called: Spermatogonial Stem Cell Transplantation, or for short: SSCT. Within this process, a patient’s cells are collected to later be given back to that same patient.	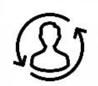<br>**Text:** “Spermatogonial Stem Cell Transplantation (SSCT)”
Introduction (3)	This figure illustrates the process. We would like to point out, that this treatment isn’t currently available. The figure depicts the process as it might occur in the future.	Overview Image 1<br>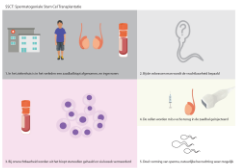
Step 1	Step 1. In the past a biopsy of the testis has been collected in the hospital, and frozen. This was done because the treatment against cancer could damage the stem cells in the testis, the so-called ‘spermatogonial stem cells’. These stem cells are important for the production of sperm cells. If too many stem cells are damaged, that person can become infertile. That’s why a small piece of the testis, containing these stem cells, is collected. This testis tissue could potentially contribute to a therapy to restore fertility in the future, to be able to have children even after treatment.	Image 1 step 1<br>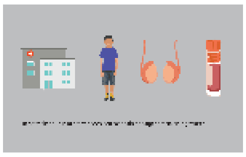
Step 2	Step 2. The fertility of the adult man is assessed. Thankfully, not all men who are treated for cancer actually become infertile. Even after a cancer treatment, there is often enough sperm production to allow for conceiving through natural conception. Sometimes there’s a decreased fertility, which can make it necessary to use additional medical reproductive treatments, like IVF, to conceive a child. And sometimes, a man is completely infertile. In that case, the cryopreserved testicular tissue could be used.	Image 1 step 2.<br>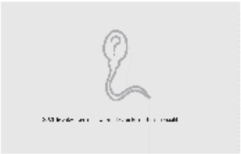
Step 3	Step 3. In case of infertility, stem cells are isolated from the biopsy and propagated in culture.<br>Within the piece of testis tissue there are spermatogonial stem cells present: progenitor cells of sperm production. The tissue from the biopsy is retrieved from storage, thawed, and the stem cells are collected. These cells are then placed in a culture dish in the lab and propagated. The number of stem cells in the testis biopsy is very small, so the purpose of culture is to increase their numbers, before placing them back in the testis of the healthy patient.	Image 1 step 3.<br>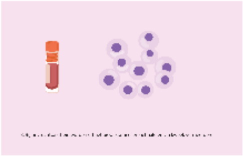

Step 4	Step 4. The cells are injected back into the testis using ultrasonic guidance.<br>If enough cells are cultured, they are inserted into the testis. The procedure is as follows: local anesthesia is applied to the patient's testis, so that it is temporarily numb. The cells are then injected into the testis, whilst ultrasound is used to make sure they end up in the right place. Because the testis is numbed, this doesn't hurt. From the injection site the stem cells then find their way back to their own, natural place within the testis, where sperm production normally takes place.	Image 1 step 4.<br>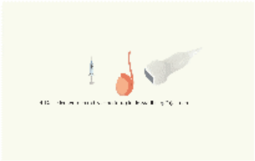
Step 5	Step 5 – The goal of the therapy: production of sperm and natural conception is possible again.<br>The transplanted stem cells can restart the process of sperm production. This takes time, so it could take months before the man produces sperm again. If enough sperm cells are produced, it would be possible to conceive a child by natural conception (through sex). If there aren't enough sperm cells to make that possible, potentially a few sperm cells could still be collected to achieve a pregnancy through use of an additional medical treatment, like IVF.	Image 1 step 5<br>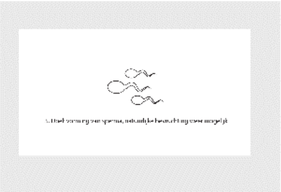
Summary of treatment	We hope this gives you a clear idea of the intended treatment. This therapy is currently not offered to patients in the hospital, because it has never been done in humans before. So, this therapy is currently in a research-phase.	Image 1 overview<br>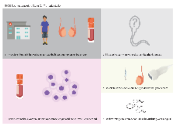
Introduction research	In the past, however, a lot of research has been done on the use of this treatment in various animal species, and the culture of human cells in the lab. We would now like to give you a brief overview of several milestones in this research history.	Image 2 overview<br>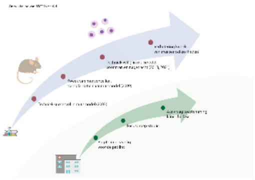
Research (1)	In 2003, researchers have shown that the culture and transplantation of the cells are effective in mice. They transplanted spermatogonial stem cells from one mouse into the testis of another, infertile mouse. The mouse who received the transplantation was after some time able to again have offspring (mouse pups).	Image 2 Step 1<br>*(NB for each consecutive step a different topic on the Image is highlighted*)<br>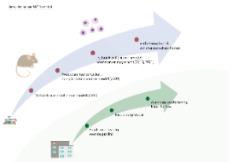
Research (2)	In 2009 a study was published that showed that the culture of spermatogonial stem cells of humans is also possible. These stem cells have not been transplanted to a human.<br>But, purely to show that the technique works, these cells were transplanted to an infertile mouse instead. This does not make the mouse fertile again – men and mice are too different for that – but the researchers were able to demonstrate that after injection the stem cells ended up in the intended proper, natural place in the testis.	Image 2 Step 2<br>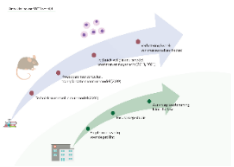

Research (3)	In 2018 and 2021, studies have been done to assess the safety of the culture and transplantation of these stem cells. Both of these studies have been done in mice.<br>The study of 2018 shows that transplanting the stam cells is not dangerous to the mouse who receives the transplantation. Injecting the cells into the testis is safe.<br>The study of 2021 shows that transplanting the stem cells is also not dangerous to the mouse pups that are born as a result. These mouse pups have been checked for their general health and their development from pup to adult. This has been done for two generations, so also the pups of the pups of the first transplanted mouse have been checked. No abnormalities were observed, compared to control mice and control-pups that were born without a prior stem cell transplantation.	Image 2 Step 3<br>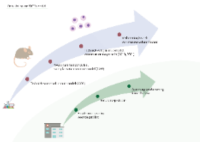
Research (4)	The studies using these mouse models show promising results for the therapy. But the treatment has not been performed in humans yet. However, in the hospital research is being done on cells from human testes. This research focusses on improving the cell culture, so as many stem cells as possible can be cultured from a small testicular biopsy, to transplant back.	Image 2 Step 4<br>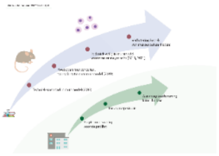
Research (5)	Previous research on human has shown, that taking the biopsy itself is safe. And that the tissue can be stored well ‘in the freezer’, in nitrogen.	Image 2 Step 5<br>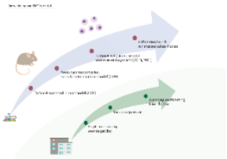
Research (6)	Other research in humans involves collecting the perspectives and thoughts of survivors of childhood cancer about taking and using the testicular biopsy. This research is done through focus groups and individual interviews. This information clip is part of this research, in which you are currently participating.	Image 2 Step 6<br>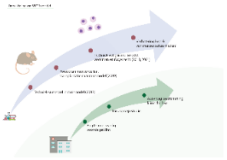
Research (7)	In the future, we want to apply for permission to take spermatogonial stem cell transplantation to the clinical phase trials. This means, that the treatment will be offered to a select group of people. These people will then be studied during the course of this treatment. In this phase, it is still an experimental therapy. If the treatment works and safe within this limited group of people, the therapy could then be implemented more widely and be offered to all men, who have a testicular biopsy stored and who would like to use it.	Image 2 Step 7<br>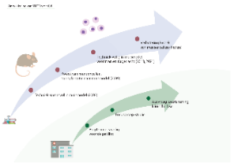
Wrap-up	This concludes what we would like to tell you about spermatogonial stem cell transplantation and the research that has been done in this field in the past, and that is currently being done. This is the end of this video.	Amsterdam UMC Logo;<br>Amsterdam UMC

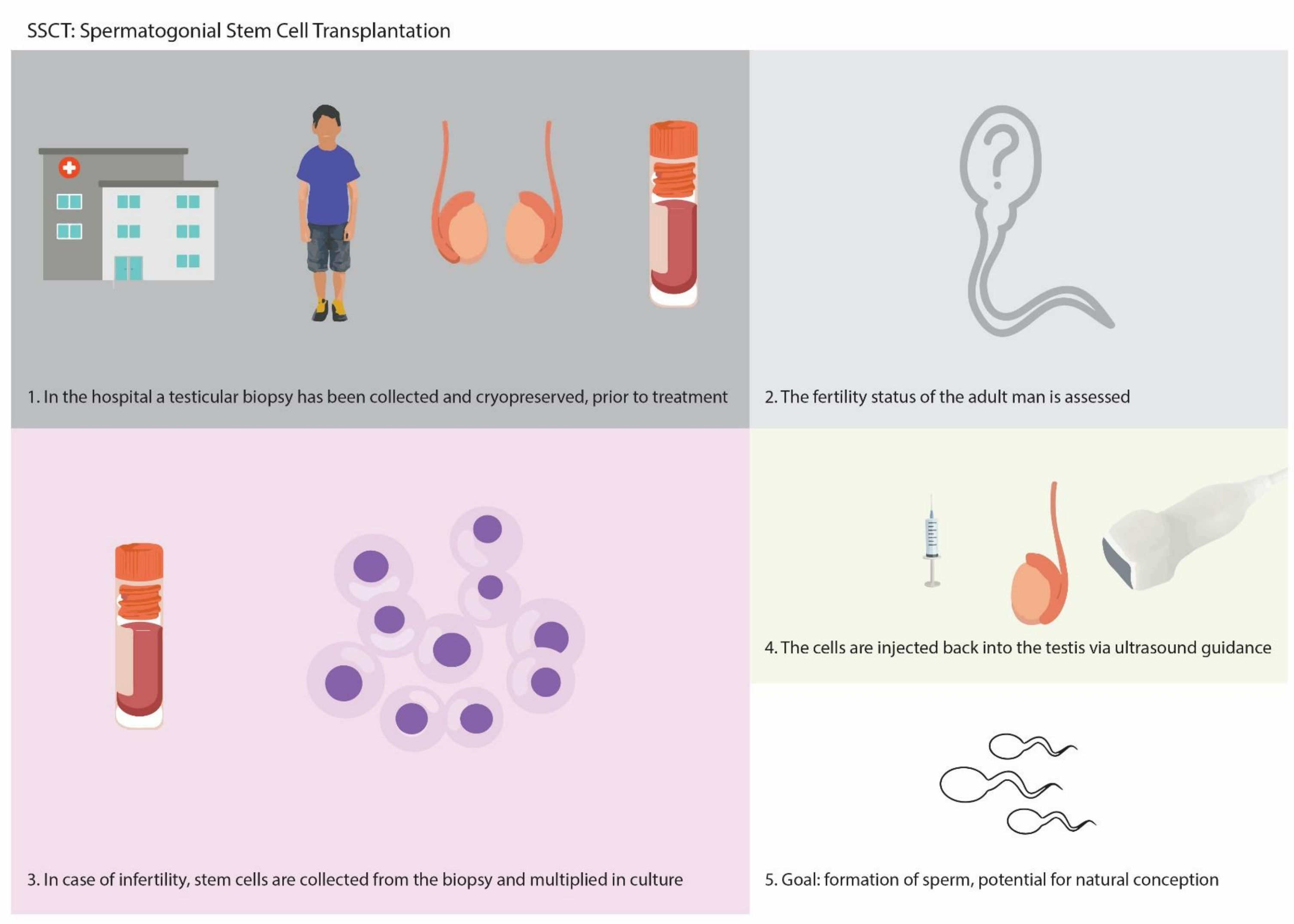

Supplementary Figure 1 - Image for use in informational video on the process of SSCT, for use during interviews. Use of various parts of the image is further illustrated in Supplementary Table 2.

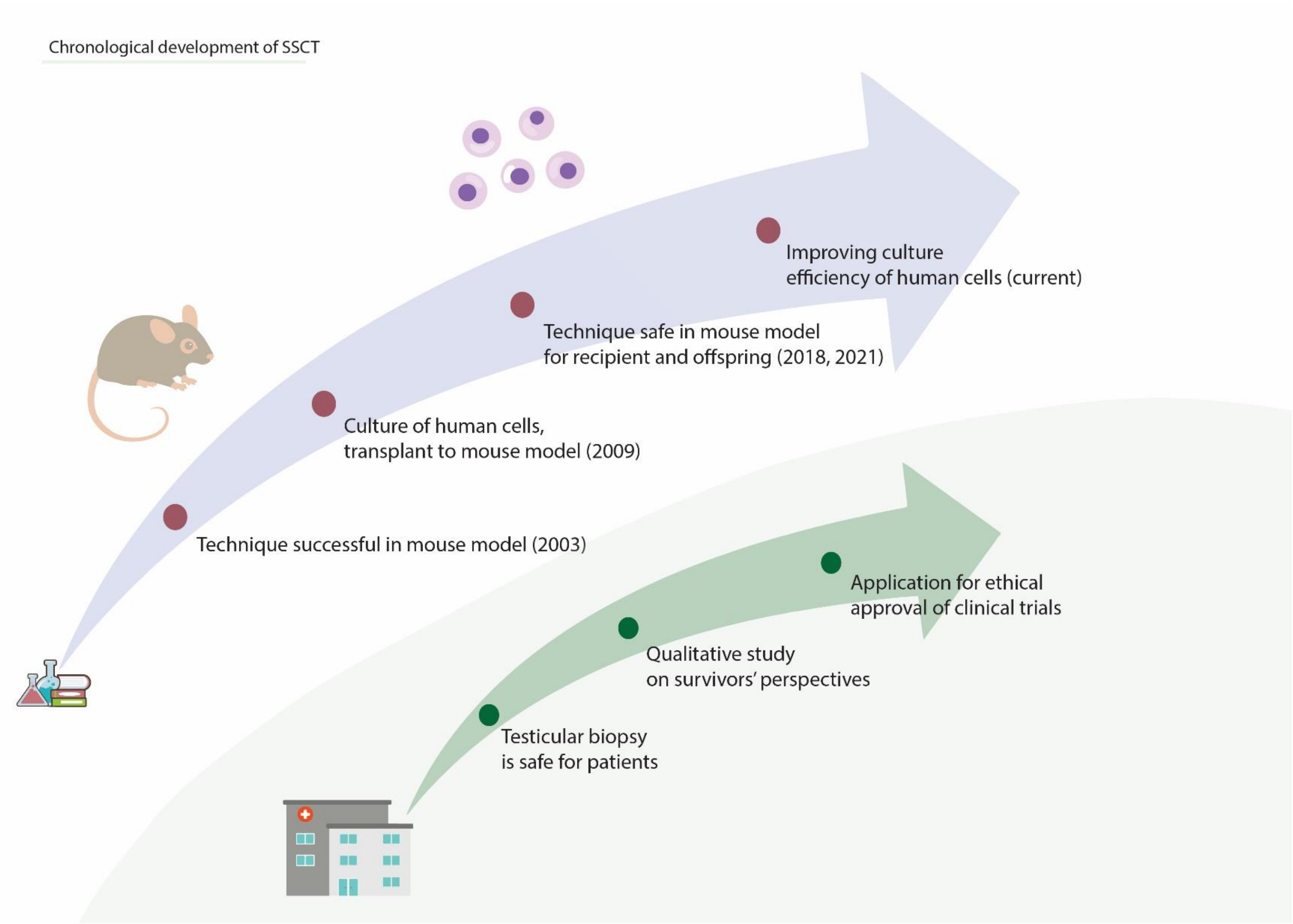

Supplementary Figure 2 - Image for use in informational video on the research on SSCT, for use during interviews. Use of various parts of the image is further illustrated in Supplementary Table 2.
